# Extracellular catalysis of environmental substrates by *Shewanella oneidensis* MR‐1 occurs via active sites on the C‐terminal domains of MtrC

**DOI:** 10.1002/pro.70243

**Published:** 2025-07-28

**Authors:** Alejandro Morales‐Florez, Colin W. J. Lockwood, Benjamin W. Nash, Marcus J. Edwards, Jessica H. van Wonderen, Amit Sachdeva, Julea N. Butt, Thomas A. Clarke

**Affiliations:** ^1^ School of Biological Sciences University of East Anglia Norwich UK; ^2^ School of Chemistry University of East Anglia Norwich UK; ^3^ School of Life Sciences University of Essex Colchester UK

**Keywords:** *c*‐type cytochrome, electron transfer, flavin, heme chain, microbe–mineral interface, *Shewanella*

## Abstract

The Gram‐negative *Shewanellaceae* family is well known for its ability to transfer catabolically derived electrons to extracellular terminal electron acceptors through electron conduits that permeate the outer membrane. The primary conduit is MtrCAB, a trimeric porin‐cytochrome complex that contains the cell surface exposed decaheme cytochrome MtrC. This donates electrons to extracellular substrates, including OmcA, soluble metals, organic electron shuttles, and insoluble metal oxides. However, it is not clear whether this broad substrate specificity requires specific sites for binding and reduction, or whether reduction occurs through non‐specific interactions near exposed hemes on the cytochrome surface. *Shewanella oneidensis* MtrC is composed of four domains, with the hemes closely packed and distributed evenly between domains II and IV. The domains are arranged to allow electron transport across the cytochrome via interdomain electron transfer, but the significance of this conserved feature is not understood. Here we use site‐directed mutagenesis to generate an MtrC variant that is comprised only of domains I and II (MtrC_DI,II_). The properties of this MtrC_DI,II_ are effectively identical to domains I and II of full‐length MtrC. Whole‐cell assays revealed that *S. oneidensis* cells replacing full‐length MtrC with MtrC_DI,II_ had significantly lower rates of OmcA, flavin mononucleotide, and Fe(III) citrate reduction. Our results demonstrate that MtrC domains III and IV contain sites for association of specific substrates, enabling the reduction of extracellular electron acceptors in *S. oneidensis*.

## 1 INTRODUCTION

To survive in environments where oxygen is limited or completely absent, many bacterial species reduce extracellular substrates using electrons produced during respiration. This requires the electrons released during intracellular metabolism to be transported from the cytoplasm to the cell surface where they can be transferred to terminal electron acceptors (Edwards et al., 2015; Richardson, 2000). Both Gram‐positive and Gram‐negative bacteria are capable of this process through the assembly of electron transfer chains that pass through the outer membrane or cell wall. Cells can reduce extracellular substrates either directly at the cell surface or indirectly via redox mediators like metal chelates or organic shuttles (White et al., 2016). The primary mechanism for extracellular electron transfer (EET) will depend on the biochemical pathways inside the bacterium, the composition of the environment, and whether the bacterium is within a biofilm or planktonic state. These variables make it challenging to predict and model the effects of microbial metabolism on minerals within the environment (Gralnick & Newman, 2007; Hernandez & Newman, 2001; Le Laz et al., 2014; Phan et al., 2024).

The *Shewanellaceae* family are facultative anaerobes renowned for their respiratory flexibility and are often used to study EET under laboratory conditions (Fredrickson et al., 2008). Most species reduce a broad range of extracellular substrates, including insoluble metal oxides, humic acids, soluble metal chelates, and synthetic textile dyes (Gralnick & Newman, 2007; Hernandez & Newman, 2001; Le Laz et al., 2014; Phan et al., 2024). In particular, *Shewanella oneidensis* MR‐1 is one of the first bacteria to have been shown to support EET and has become a model system for understanding EET in Gram‐negative bacteria.

**FIGURE 1:**
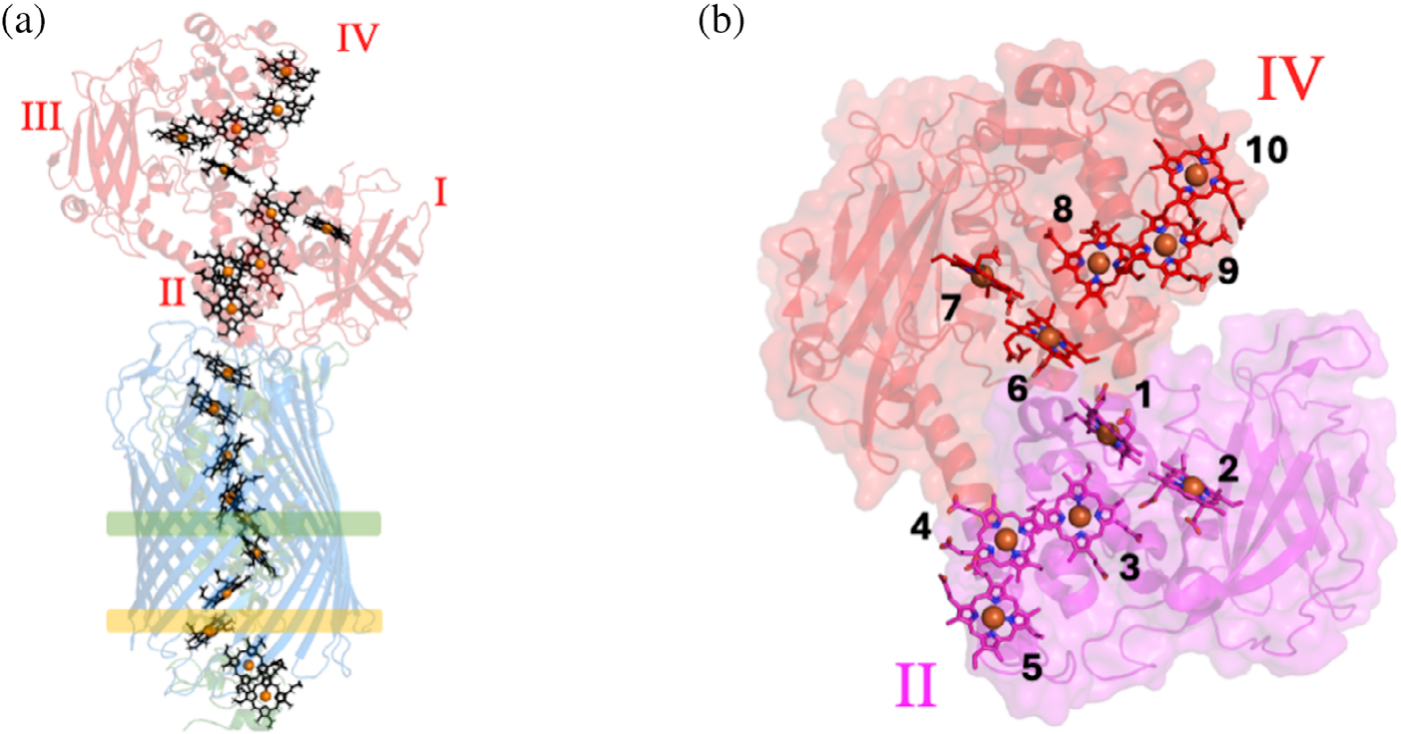
X‐ray crystal structure of the MtrCAB complex. (a) Cartoon representation of the complex from *Shewanella baltica* OS185 (PDB: 62RQ). MtrA (green) which is surrounded by MtrB (blue). MtrC (red) is associated to the extracellular side of MtrB. The four domains of MtrC are indicated in roman numerals. The predicted positioning of the periplasmic (yellow box) and extracellular (green box) faces of the lipid bilayer are shown. (b) Staggered cross heme arrangement within MtrC from *S. oneidensis* MR‐1 (PDB: 4LM8) showing hemes distributed evenly across two pentaheme domains. Hemes 1–5 are found within domain II (pink), and hemes 6–10 within domain IV (red). The hemes are numbered according to the position of the heme binding motif within the corresponding polypeptide chain.

All *Shewanella* capable of EET contain an *mtr* gene cluster that encodes for MtrCAB, a porin‐cytochrome complex that transports electrons across the outer membrane and reduces extracellular electron acceptors (Fredrickson et al., 2008). An ~80 Å transmembrane heme chain is formed by the decaheme cytochrome, MtrA, enveloped by the outer membrane porin MtrB. An outer membrane cytochrome (OMC) termed MtrC binds to the surface of MtrAB and can directly transfer electrons to extracellular terminal acceptors (Figure 1a) (Edwards et al., 2020; White et al., 2013).

In addition to the core *mtrCAB* genes, the *mtr* gene cluster also typically contains a range of other genes encoding for additional OMCs and porin‐cytochrome complexes. In *S. oneidensis* MR‐1 these include *omcA*, which encodes a second cell surface localized decaheme OMC, and *mtrDEF* that encodes a second porin‐cytochrome complex (Coursolle & Gralnick, 2010; Lockwood et al., 2018). Extensive studies have been undertaken to better understand how these cytochromes contribute to the interactions between *S. oneidensis* MR‐1 and different extracellular electron acceptors. These include: insoluble metal oxides; flavins; soluble and chelated metal ions; humic acid analogs; electrodes and azo dyes (Bencharit & Ward, 2005; Brutinel & Gralnick, 2012; Cai et al., 2012; Hong et al., 2007; Le Laz et al., 2014; Thompson et al., 2002; Wu et al., 2009). Overall, these studies indicate that the roles of MtrC and OmcA overlap heavily, particularly with the reduction of soluble substrates. For insoluble metals there is evidence that OmcA and MtrC interact differently with different minerals, but these studies cannot discern whether these differences are due to differences in organization and orientation on the surface, or differences in the structures of the two OMCs (Jing et al., 2020). Consequently, the rationale for the production of different OMCs on the cell surface remains unclear.

The overall structures of different OMCs are largely conserved. Most OMCs contain a core of 10 hemes arranged in a “staggered cross” formation within a four‐domain structure consisting of a repeated β‐barrel–cytochrome motif (Figure 1b) (Edwards et al., 2015, 2020). In both MtrC and OmcA, the first five hemes are located within domain II, and hemes 6–10 are within domain IV. The split β‐barrels in domains I and III are arranged to enable interactions between domain II and domain IV that allow electron exchange between all 10 hemes (Edwards et al., 2020; Norman et al., 2023). All hemes of OMCs identified so far are histidine coordinated, and there are no obvious sites for binding and subsequent reduction of substrates. This makes it unclear how these OMCs can catalyze the reduction of such a broad range of substrates.

Here, we explore the conserved duplication of the β‐barrel–cytochrome motif in MtrC and its impact in both reduction of soluble extracellular substrates and electron transfer partners. Site‐directed mutagenesis allowed the production of a truncated MtrC variant comprised solely of domains I and II (MtrC_DI,II_) that is near‐identical to domains I and II of the full‐length MtrC. This truncated form retains its ability to interact with MtrAB but is unable to reduce key environmental substrates, including flavin mononucleotide (FMN) and Fe(III) citrate. These results suggest that MtrC has evolved discrete sites for binding and reduction that are largely formed by the presence of MtrC domains III and IV.

## 2 MATERIALS AND METHODS

### 2.1 Bacterial strains, mutants, plasmids, and growth conditions

*S. oneidensis* MR‐1, *S. oneidensis* Δ*mtr*, and *S. oneidensis* Δ*mtrC/omcA* were used as described previously (Jing et al., 2020). For characterization of soluble MtrC variants, a pBAD202/D‐TOPO expression vector containing the *kanR* gene and *mtrC* with an *mtrB* signal peptide without a lipid anchor (pJvW001) was used (Table S1) (Lockwood et al., 2018, 2024). A codon encoding glutamic acid at amino acid position 344 was replaced by a premature stop codon in pJvW001, resulting in plasmid pAMF1 (Tables S1 and S2). pAMF1 was transformed via electroporation into *S. oneidensis* MR‐1. The pBAD202/D‐TOPO plasmid pLS138 contains the native *mtrC* gene encoding a recombinant MtrC associated with the outer membrane by a lipid anchor (MtrC_memb_). The stop codon was introduced to pLS138 at the same position in the *mtrC* gene, resulting in the pAMF2 plasmid (Table S1). The pAMF2 plasmid was transformed via electroporation into *S. oneidensis* Δ*mtrC/omcA*.

### 2.2 Purification of MtrC_DI_ _,II,sol_

*S. oneidensis* MR‐1 cells containing pAMF1 (encoding for soluble MtrC_DI,II__,_ or MtrC_DI,II,sol_) were cultured and induced with 5 mM L‐arabinose in 1 L flasks in M72 media with kanamycin (30 μg mL^−1^) as described previously (Li et al., 2020). The cells were harvested by centrifugation (30 min, 5500 × *g*, 4°C) 20 h after induction. Supernatant was concentrated using tangential flow filtration (Vivaflow™ 200, 10,000 molecular weight cut‐off [MWCO]) to 100 mL and further concentrated using a centrifugal concentrator (Vivaspin® 10,000 MWCO) to 200 μL. The concentrate was diluted in 500 mL of 20 mM Tris, 30 mM NaCl, pH 7.8 before running on a diethylaminoethyl (DEAE) chromatography column eluted using a 0–0.5M NaCl gradient. Protein purity was assessed by sodium dodecyl sulfate polyacrylamide gel electrophoresis (SDS‐PAGE), as described previously (Edwards et al., 2020). Fractions containing MtrC_DI,II,sol_ were pooled and concentrated using a centrifugal concentrator (Vivaspin® 10,000 MWCO) to 1 mL. The concentrated protein was applied to a HiLoad® 16/600 Superdex® 75 pg size‐exclusion chromatography column equilibrated with 100 mM Tris, 150 mM NaCl, pH 8.1, and eluted in the same buffer at 0.5 mL min^−1^, 4°C. SDS‐PAGE was used to identify fractions containing MtrC_DI,II,sol_ (Figure S2), which were subsequently pooled and concentrated using a centrifugal concentrator (Vivaspin® 10,000 MWCO) before storage at −80°C.

### 2.3 Purification of MtrC_DI,II_AB complex

*S. oneidensis* Δ*mtrC/omcA* cells containing pAMF2 (encoding for the lipid‐anchored MtrC_DI,II,_ or MtrC_DI,II,memb_) were cultured as above except that cells were grown at 25°C and gene expression was induced with 1 mM L‐arabinose. The MtrC_DI,II_AB complex was purified as described previously (Edwards et al., 2020). Fractions containing MtrC_DI,II_AB were identified via SDS‐PAGE and subsequently pooled and concentrated using a centrifugal concentrator (Vivaspin® 100,000 MWCO) to 1 mL. In addition to the previously published protocol, the protein was loaded onto a Mono‐Q chromatography column pre‐equilibrated with Buffer A (20 mM 4‐(2‐hydroxyethyl)‐1‐piperazineethanesulfonic acid (HEPES), 5 mM lauryldimethylamine oxide (LDAO), pH 7.8) and eluted with a 0–0.5M gradient of NaCl in Buffer A, at 0.5 mL min, 4°C. Fractions containing MtrC_DI,II_AB were identified via SDS‐PAGE and subsequently pooled and concentrated using a centrifugal concentrator (Vivaspin® 100,000 MWCO).

### 2.4 UV–visible spectroscopy

Samples were diluted in 500 μL of 20 mM Tris, 30 mM NaCl, pH 7.8, and the UV–visible spectra were recorded using a CARY 60 UV–visible spectrophotometer (Agilent). To fully reduce MtrC_DI,II,sol_, excess sodium dithionite was added (2 μL at 1 mg mL^−1^ from an anaerobic stock prepared in Milli‐Q water). The concentration of oxidized MtrC_DI,II,sol_ was quantified using a predicted extinction coefficient at 410 nm (*ε*_410nm_) of 663 mM^−1^ cm^−1^, which corresponded to half the full‐length MtrC extinction coefficient reported previously (van Wonderen et al., 2021).

### 2.5 Liquid chromatography‐mass spectrometry

Protein samples were prepared in 20 mM Tris, 5 mM NaCl, pH 7.5. Fifty micromolar protein was diluted 20‐fold into 0.3% (v/v) formic acid and 1% (v/v) acetonitrile. The samples were run through a ProSwift RP‐1S column (4.6 × 50 mm, Thermo Scientific™) on an Ultimate 3000 uHPLC system (Dionex, Leeds, UK). Samples were eluted on a linear gradient of 2%–100% acetonitrile, 0.1% formic acid. A Bruker microQTOF‐QIII mass spectrometer was used to carry out positive mode electrospray ionization mass spectrometry.

### 2.6 X‐ray crystallography

MtrC_DI,II,sol_ crystals were obtained from a sitting‐drop vapor diffusion setup in a 96‐well 1‐drop crystallization plate with 0.2M magnesium acetate tetrahydrate, 0.1M sodium cacodylate, 20% w/v polyethylene glycol (PEG) 8000, pH 6.5 as the reservoir solution. Total drop volume was 0.5 μL with a 1:1 (protein:reservoir). Crystals were cryoprotected by transfer into 0.2M magnesium acetate tetrahydrate, 0.1M 2‐morpholinoethanesulphonic acid, 20% w/v PEG 8000, 20% ethylene glycol, pH 6.5, before vitrification by plunging into liquid nitrogen. The data were collected from beamline I24 at Diamond Light Source (Table S3). The average cell dimensions of the crystals were *a* = 74.36 Å, *b* = 77.15 Å, *c* = 96.52 Å, with space group P2_1_2_1_2_1_. Data were collected using an x‐ray wavelength of 0.9999 Å and processed using xia2 DIALS (Winter, 2010) and AIMLESS (Evans & Murshudov, 2013). A molecular replacement model was generated by removing domains III and IV of MtrC (PDB: 4LM8). This model was refined using Phenix (Liebschner et al., 2019) and Refmac5 (Murshudov et al., 2011) to a final resolution of 1.8 Å. Coordinates have been added to the Research Collaboratory for Structural Bioinformatics Protein Data Bank with accession code 9EOV.

### 2.7 Protein film electrochemistry

Protein film electrochemistry was performed in 50 mM HEPES, 100 mM NaCl at pH 7.0 using hierarchically structured mesoporous indium‐tin oxide (ITO) working electrodes (0.25 cm^2^ surface area, 20 μm thickness) deposited on glass slides coated with fluoride‐doped tin oxide, as described previously (Jenner et al., 2022; Mersch et al., 2015). An electrode was chilled to 4°C (20 min on ice) before the protein (40 μM protein in 100 mM Tris, 150 mM NaCl, pH 8.1) was applied to the electrode and left to adsorb for 15 min. Cyclic voltammetry was performed in a 3‐electrode configuration with a Pt wire counter electrode and Ag/AgCl (saturated with KCl) reference electrode. The electrochemical cell was housed in a Faraday cage within a N_2_‐filled chamber (atmospheric O_2_ < 2 ppm). Measured potentials were converted to standard hydrogen electrode (SHE) by addition of 0.195 V.

### 2.8 Analytical ultracentrifugation

Sedimentation velocity analyses were performed on purified MtrC_DI,II_AB, MtrAB, and MtrCAB at an Abs_410nm_ ~ 0.7 in 20 mM HEPES, 150 mM NaCl, 5 mM LDAO, pH 7.8. Centrifugation was carried out at 128,794 × *g*, 20°C, and an Abs_410nm_ scan was recorded every 3 min (200 scans). SEDNTERP was used to calculate a buffer viscosity and density of 1.0305 × 10^−2^ P and 1.00602 g mL^−1^, respectively (Philo, 2023). The partial specific volume was unchanged and left at 0.730 mL g^−1^, and the *f*/*f*_0_ ratio was fitted to 1.56 for each analysis. The data were fitted in SEDFIT using *c*(*s*) and *c*(*M*) distribution analysis (Li et al., 2020; Schuck, 2000).

### 2.9 Measurements of cellular reduction of extracellular electron acceptors

For all measurements, cells were prepared similarly. Ten milliliters of Luria broth (LB) was inoculated with cells picked from single *S. oneidensis* colonies on LB agar plates and incubated aerobically at 30°C until the cell culture reached OD_600nm_ ~1.0. Afterward, 10 mL of M72 media with supplements (Li et al., 2020), kanamycin (30 μg mL^−1^), and L‐arabinose (1 mM) was inoculated with 0.1% of the cell culture, sealed, and incubated (18 h, 25°C, 120 RPM) to become microaerobic. Next, cultures were transferred into an anaerobic chamber where they were opened for 1 h to remove any residual oxygen. The cells were sealed and harvested by centrifugation (10 min, 3000 × *g*, 21°C) before resuspension in sterile solution comprised of anaerobic *Shewanella* minimal medium (*Shewanella* basal medium, 100 mM HEPES, vitamin mix 2.5 mL L^−1^, and mineral mix 2.5 mL L^−1^, pH 7.2, prepared in Milli‐Q water (Baron et al., 2009)) supplemented with fresh, anaerobic 20 mM sodium DL‐lactate Shewanella minimal media (SMM)_lactate_. All measurements of the cellular reduction of extracellular electron acceptors were performed in SMM_lactate_ solutions at an OD_600_ of 0.1.

To measure rates of FMN reduction, an anaerobic stock solution of 1 mM FMN in Milli‐Q water, pH 7, was added to a sealed 3 mL fluorescence cuvette containing *S. oneidensis* cells resuspended in SMM_lactate_, so the final FMN concentration was 12 μM. Fluorescence was recorded immediately upon addition of FMN at an excitation of 365 nm and emission of 525 nm (Cary Eclipse Fluorescence Spectrophotometer, Agilent).

To measure rates of OmcA reduction, OmcA was prepared as reported previously (Edwards et al., 2014). Seven hundred nanomolar of OmcA was added to cells in a 96‐well plate to a final volume of 250 μL under anaerobic conditions. The plate was sealed inside the anaerobic chamber with an adhesive film and was further sealed by applying polystyrene cement to the plate lid. Absorbance measurements at 409 nm were recorded in a plate reader using a path length of 7.89 mm (FLUOstar Omega, BMG LABTECH). The *ε*_409nm_ = 1670 mM^−1^ cm^−1^ for oxidized OmcA (Ross et al., 2009) was used to determine the concentration of oxidized OmcA. After the experiment, excess sodium dithionite was added to provide the spectrum of fully reduced OmcA so changes in Abs_552nm_ could be converted to the proportion of OmcA that was reduced or remained oxidized.

To measure rates of azo dye reduction, anaerobic stock solutions were prepared of Reactive Black 5 (RB5), Amaranth, and Methyl Orange (all 1 mM in Milli‐Q water). Anaerobic 96‐well assay plates were prepared as above with final concentrations of RB5, Amaranth, or Methyl Orange being 60, 30, or 60 μM. Reduction rates for each azo dye were calculated using their extinction coefficients: RB5 (*ε*_600_ = 20 mM^−1^ cm^−1^), Amaranth (*ε*_520_ = 22.6 mM^−1^ cm^−1^), and methyl orange (*ε*_464_ = 21.6 mM^−1^ cm^−1^) (Bissaro et al., 2022; Blümel et al., 2002; Saraswati et al., 2018).

To measure rates of Fe(III) citrate and Fe(III) ethylenediaminetetraacetic acid (EDTA) reduction, *S. oneidensis* cells were resuspended in SMM_lactate_ supplemented with 1 mM L‐arabinose and 5 mM Fe(III) citrate or Fe(III) EDTA. Twenty‐five milliliters of samples were prepared in universal 25 mL containers and sealed with a Suba‐Seal®, resulting in a reduced headspace, and incubated at 30°C, 0 RPM. To quantify Fe(III) iron reduction, 750 μL samples were extracted with a sterile needle through the Suba‐Seal® and centrifuged (5 min, 20,000 × *g*, 21°C) to remove insoluble species. Five hundred microliters of supernatant was incubated for 1 min with 30 μL of FerroZine™ solution (10 mM 3‐(2‐pyridyl)‐5,6‐diphenyl‐1,2,4‐triazine‐4′,4″‐disulfonic acid, 100 mM ammonium acetate) and the Abs_562nm_ was measured. Standard curves were produced using ferrous chloride dissolved in water.

## 3 RESULTS

### 3.1 Assembly and characterization of a soluble truncated pentaheme MtrC

**FIGURE 2:**
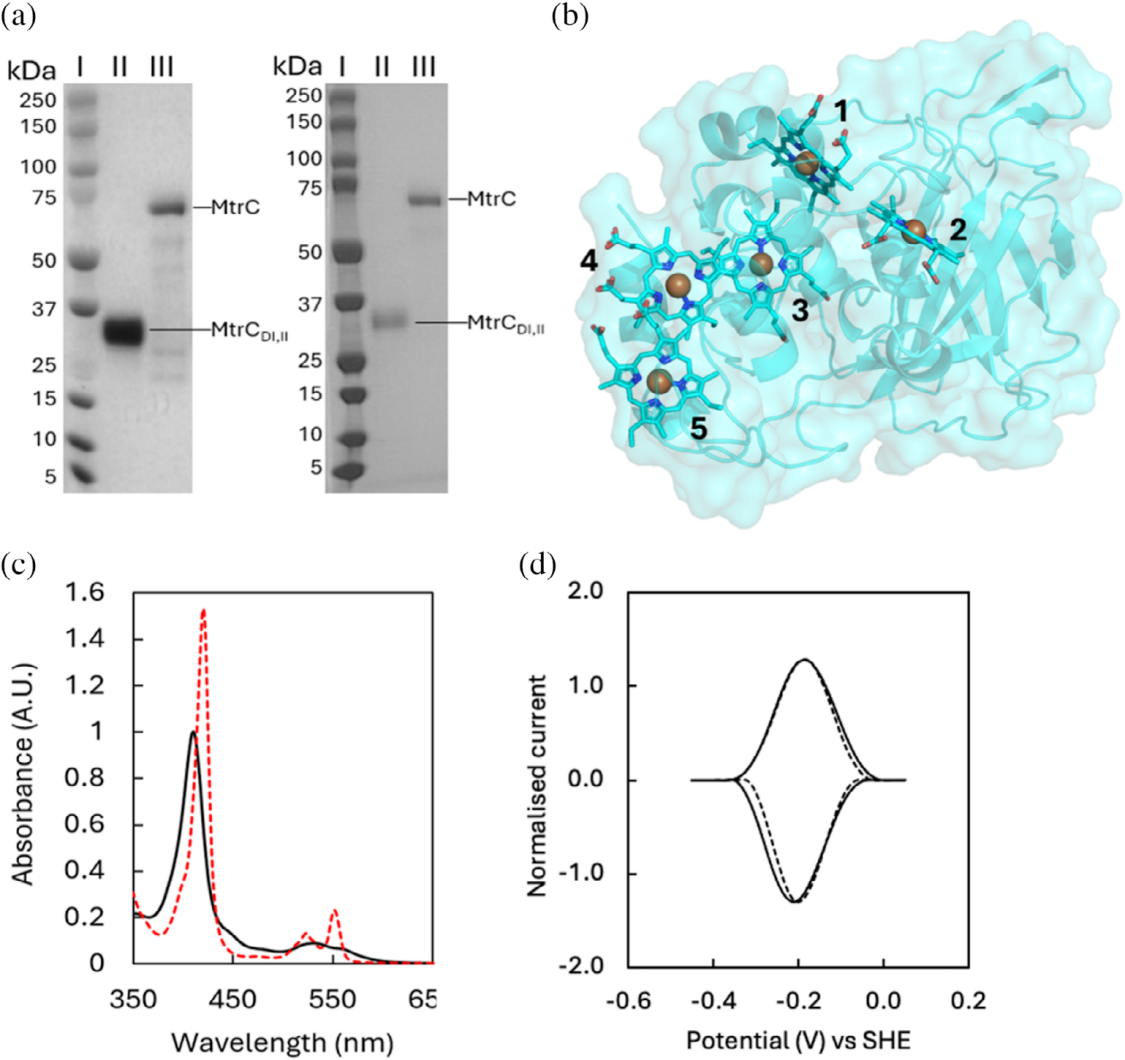
Purification and structural characterization of MtrC_DI,II_. (a) SDS‐PAGE gel images of purified MtrC_sol_ and MtrC_DI,II,sol_, visualized by peroxidase‐linked heme stain (left) and Coomassie stain (right). Molecular weight marker (lane I), purified MtrC_DI,II,sol_ (lane II) and MtrC_sol_ (lane III). (b) x‐ray diffraction crystal structure of MtrC_DI,II,sol_ (PDB: 9EOV) at 1.8 Å resolution. Hemes are numbered according to position of the heme‐binding motif within the amino acid chain. (c) Electronic absorbance of MtrC_DI,II,sol_ in the oxidized state (solid black line) and fully dithionite‐reduced state (red dashed line). (d) Protein film voltammetry of MtrC variants. MtrC_DI,II,sol_ and MtrC_sol_ are shown as solid and dashed lines respectively. Buffer electrolyte solution was composed of anaerobic 50 mM HEPES, 100 mM NaCl, pH 7.0. Cyclic voltammetry was at 20 mV s^−1^ with protein adsorbed on mesoporous hierarchical indium‐tin oxide electrodes. SHE, standard hydrogen electrode.

This study initially assessed the ability of the N‐terminal domains I and II of MtrC to retain their structural integrity and bind five *c*‐type hemes in the absence of the C‐terminal domains III and IV. The pAMF1 plasmid (encoding for MtrC_DI,II,sol_) was transformed into *S. oneidensis* MR‐1 and used to produce MtrC_DI,II,sol_ as described in the methods. SDS‐PAGE gels stained for heme revealed the isolated species was a single *c*‐type cytochrome with a mass of ~35 kDa, approximately half that of MtrC_sol_ (Figure 2a). Liquid‐chromatography mass spectrometry (LC–MS) analysis of the purified protein showed several minor peaks with masses between 34,962 and 35,466 Da, with a major peak at 35,364 Da (Figure S2). The minor peaks closely match the predicted masses of MtrC variants with C‐terminal residues between positions 321–326. The major peak is close to the predicted mass of 35,361 Da (accounting for a mass of 615.17 Da per heme; Yang et al., 2005) for MtrC having Ala325 as the C‐terminal residue.

The electronic absorbance of the sample was measured under air‐equilibrated (i.e., oxidized) and fully sodium dithionite‐reduced states (Figure 2c). The spectrum of the oxidized sample contained peaks with maxima at 410 and 525 nm, consistent with the Soret, α, and β features of Fe(III)‐containing *c*‐type hemes (van Wonderen, Morales‐Florez, et al., 2024). Upon sample reduction, there was a shift in the Soret peak maximum from 410 to 420 nm and a sharpening of the α and β bands (maxima at 552 and 525 nm, respectively). These spectral features are typical of redox‐active, low‐spin, bis‐histidine coordinated *c*‐type hemes, and together with the LC–MS, indicate the protein consists of a soluble MtrC pentaheme cytochrome mainly truncated at amino acid residue 325. That protein is termed MtrC_DI,II,sol_ in the following text.

X‐ray crystallography was used to determine the molecular structure of MtrC_DI,II,sol_ to a resolution of 1.8 Å (Figure 2b). The structure confirmed the successful assembly of MtrC_DI,II,sol_, including the covalent attachment of five bis‐histidine coordinated *c*‐type hemes. Superposition of the MtrC_DI,II,sol_ crystal structure with the MtrC_sol_ crystal structure (PDB ID: 4LM8) yielded a root mean square displacement (RMSD) of 0.380 Å, indicating that the structure of domains I and II was not significantly affected by the absence of domains III and IV (Figure S3). The orientation of the 10 histidine imidazole rings that form the axial ligands to the heme irons is also highly conserved (RMSD of 0.225 Å), suggesting that the environment of the five hemes was unchanged.

The redox properties of MtrC_DI,II,sol_ were studied using protein film electrochemistry. MtrC_DI,II,sol_ adsorbed onto a mesoporous ITO electrode showed peaks for protein reduction (negative current) and oxidation (positive current) (Figure 2d). The redox activity of MtrC_DI,II,sol_ at pH 7 was fully reversible over an electrochemical potential window from −400 to 0 mV versus SHE. The redox potential window of MtrC_DI,II,sol_ was similar to the observed window for MtrC_sol_ measured under comparable conditions (Figure 2d). Thus, there is no evidence that either the N‐ or the C‐terminal domain of the full‐length protein holds the majority of hemes with the more negative reduction potentials.

### 3.2 MtrC_DI_ _,II_ forms part of a transmembrane electron transport complex

**FIGURE 3:**
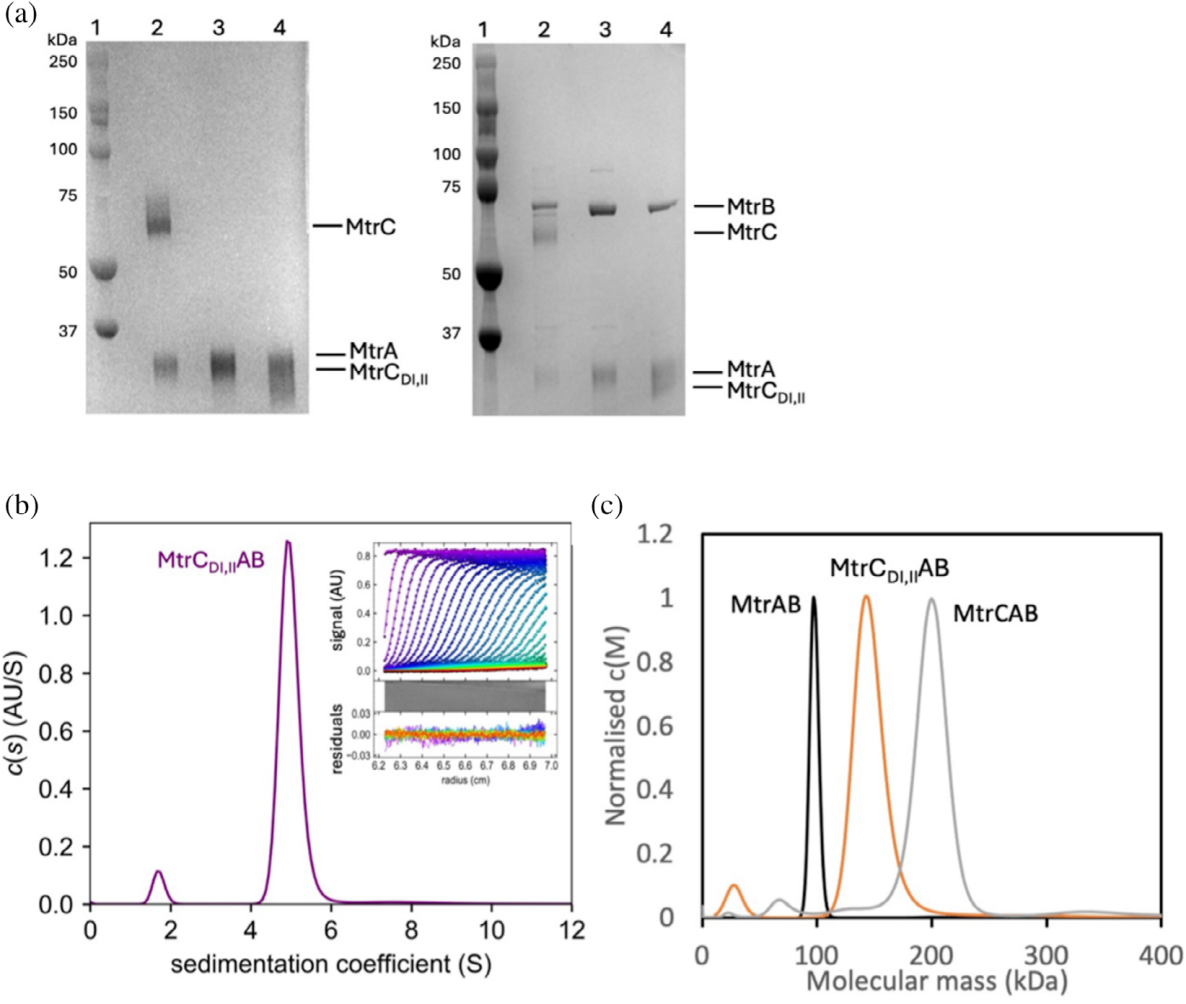
Characterization of MtrC_DI,II,memb_ in complex with MtrAB. (a) Purified protein SDS‐PAGE gels visualized by peroxidase‐linked heme stain (left) and Coomassie stain (right). Molecular weight marker (lane 1), MtrCAB (lane 2), MtrAB (lane 3), and MtrC_DI,II_AB (lane 4). (b, left) Sedimentation coefficient distribution of MtrC_DI,II_AB in 20 mM 4‐(2‐hydroxyethyl)‐1‐piperazineethanesulfonic acid (HEPES), 150 mM NaCl, 5 mM LDAO, pH 7.8. Inset: MtrC_DI,II_AB sedimentation velocity analysis observed by Abs_410nm_ (markers). Data was fit (lines) to Lamm equation and any fitted data residual absorption is shown in the lower panel. (c, right) Overlay of normalized molecular mass distributions *c*(*M*) of MtrAB (black line), MtrC_DI,II_AB (orange line), and MtrCAB (gray line).

The biophysical, structural, and spectroscopic evidence showed that the MtrC_DI,II,sol_ pentaheme cytochrome was structurally homologous to the equivalent domains of MtrC. To determine whether an MtrC_DI,II_ variant could still interact with MtrA and MtrB, it was necessary to generate a recombinant MtrC_DI,II_ that contained the N‐terminal lipid anchor associated with native MtrC (MtrC_DI,II,memb_). The pAMF2 plasmid (encoding for MtrC_DI,II,memb_) was transformed into a *S. oneidensis* Δ*mtrC/omcA* mutant (resulting in *S. oneidensis* Δ*mtrC/omcA* MtrC_DI,II,memb_) and induced as described in the methods. The complex from *S. oneidensis* Δ*mtrC/omcA* MtrC_DI,II,memb_ (denoted *S. oneidensis* MtrC_DI,II_AB) was isolated as described in Section 2. SDS gels that were stained for heme and by Coomassie revealed that all complexes contained bands at the approximate molecular weights corresponding to MtrA (38.6 kDa) and MtrB (75.5 kDa), but bands corresponding to full‐length MtrC (75.5 kDa) were only observed in the complex isolated from *S. oneidensis* MR‐1 (Figure 3a), corresponding with previous studies showing the Mtr proteins on SDS gels (Hartshorne et al., 2009; van Wonderen, Crack, et al., 2024). However, the low molecular weight heme‐containing band from *S. oneidensis* Δ*mtrC/omcA* MtrC_DI,II,memb_ was broader than the corresponding bands in the other samples, consistent with this band containing both MtrA and MtrC_DI,II,memb_ (35.4 kDa). These polyacrylamide gels are consistent with the isolation of three complexes: MtrAB, MtrCAB, and MtrC_DI,II_AB from strains *S. oneidensis* Δ*mtrC/omcA*, MR‐1, and *S. oneidensis* Δ*mtrC/omcA* MtrC_DI,II,memb_, respectively.

Each purified complex was analyzed using sedimentation velocity. Data were fitted using SEDFIT to obtain continuous distribution profiles of sedimentation coefficients (*S*) and masses (*M*) for the MtrAB, MtrC_DI,II_AB, and MtrCAB complexes. These analyses gave experimental solution masses of 99 ± 4, 144 ± 14, and 199 ± 15 kDa, respectively (Figure 3b and Table S4). These values match reasonably well with the predicted masses of 114, 149, and 185 kDa for MtrAB, MtrC_DI,II_AB, and MtrCAB, respectively. LDAO micelles have a density of 0.996 g mL^−1^, making them slightly buoyant in solution. The LDAO micelle ring around the MtrB subunit could therefore account for the differences in observed molecular masses. Taken together, the SDS‐PAGE and sedimentation data indicated that MtrC_DI,II,memb_ was able to form a stable complex with MtrAB.

### 3.3 *In vivo* reduction of physiological and non‐physiological substrates of *S. oneidensis* strains

The preceding experiments had revealed that it was possible to assemble a stable complex of MtrA, MtrC_DI,II_, and MtrB in *S. oneidensis* Δ*mtrC/omcA* MtrC_DI,II,memb_. To better understand the role of MtrC domains III and IV in substrate catalysis, the *in vivo* reduction activity of the strains *S. oneidensis*: MR‐1, Δ*mtr*, Δ*mtrC/omcA*, Δ*mtrC/omcA* MtrC_memb_, and Δ*mtrC/omcA* MtrC_DI,II,memb_ were compared using different physiological and synthetic substrates. These strains were all grown overnight under identical conditions before the addition of substrates as described in Section 2.

**FIGURE 4:**
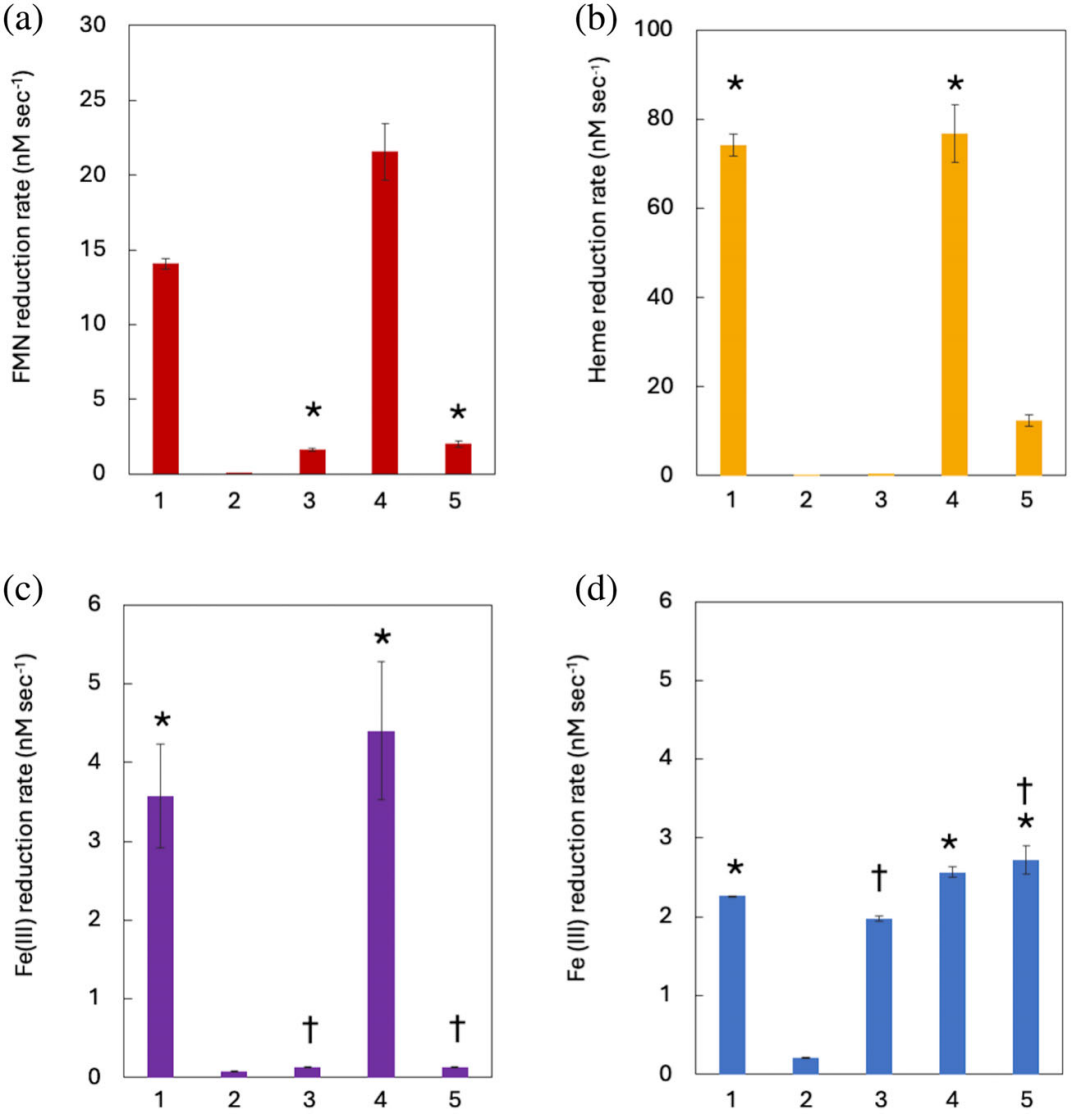
Cellular reduction of natural electron acceptors *Shewanella oneidensis* strains MR‐1 (1), ∆*mtr* (2), ∆*mtrC/omcA* (3), ∆*mtrC/omcA* MtrC_memb_ (4), and Δ*mtrC/omcA* cells MtrC_DI,II,memb_ (5) were incubated with different terminal electron acceptors. (a) Flavin mononucleotide (FMN), (b) oxidized OmcA, (c) Fe(iii) citrate, and (d) Fe(III) ethylenediaminetetraacetic acid (EDTA) reduction assays with *S. oneidensis* cells. All assays were performed with cells at a starting OD_600_ of 0.1. Rates were calculated from the change of fluorescence (absorbance) units over a period of: 15 min (a), 45 min (b), and 120 min (c, d). Experiments performed in triplicate, and error bars show the standard error of the mean. Data were analyzed using an independent *t*‐test. Distinct symbols (*, †) were used to denote separate data sets, within which rates did not differ significantly (*p* < 0.05) from one another.

The ability of the different strains to reduce the physiologically relevant substrate FMN was first investigated (Figure 4a). Both *S. oneidensis* MR‐1 and *S. oneidensis* Δ*mtrC/omcA* MtrC_memb_ were able to reduce FMN in solution, with *S. oneidensis* Δ*mtrC/omcA* MtrC_memb_ having the highest observed activity. This may be attributed to the higher concentrations of MtrC observed in this strain, as a consequence of the protein overexpression system. Strains lacking the ability to express the full‐length *mtrC* were substantially affected in their ability to reduce FMN, revealing that MtrC_DI,II,memb_ was unable to reduce FMN effectively.

The soluble Fe(III) chelates, Fe(III) citrate and Fe(III) EDTA, were next investigated (Figures 4c and 3d, respectively). Citrates are secreted by plants and microorganisms to form a range of Fe(III) citrate complexes in soils and sediments, and so are likely potential physiological substrates of *S. oneidensis* MR‐1. In contrast, EDTA is a synthetic chelator of cations, and so Fe(III) EDTA would be a non‐physiological substrate. Both *S. oneidensis* MR‐1 and *S. oneidensis* Δ*mtrC/omcA* MtrC_memb_ could reduce soluble Fe(III) citrate, while strains producing the truncated form of MtrC reduced Fe(III) citrate poorly (Figure 4b). These findings replicated the pattern observed for the reduction of FMN. Surprisingly, *S. oneidensis* Δ*mtrC/omcA* was able to reduce Fe(III) EDTA at the same rate as MR‐1, suggesting that the extracellular cytochromes MtrC and OmcA were not required for the effective reduction of the synthetic Fe(III) chelate. The reduction rates of Fe(III) EDTA by two other strains (Table S1), *S. oneidensis* Δ*mtr* MtrC_memb_ (0.2 ± 0.03 nM s^−1^) and *S. oneidensis* Δ*mtr* MtrC_DI,II,memb_ (0.13 ± 0.02 nM s^−1^) were similar to *S. oneidensis* Δ*mtr* (0.21 ± 0.01 nM s^−1^), indicating that MtrAB is required for reduction of Fe(III) EDTA.

Addition of soluble OmcA to *S. oneidensis* MR‐1 and *S. oneidensis* Δ*mtrC/omcA* MtrC_memb_ resulted in an increase in absorbance at 552 nm corresponding to the reduction of OmcA with electrons delivered from the whole cells (Figure 4b). In contrast, both Δ*mtr* and Δ*mtrC/omcA* strains were incapable of reducing soluble OmcA. *S. oneidensis ΔmtrC/omcA cells* producing MtrC_DI,II_AB reduced OmcA at an almost four‐fold lower rate when compared with *S. oneidensis* MR‐1. These results suggest that soluble OmcA can interact and receive electrons from MtrC, and that these interactions principally involve MtrC domains III and IV. Limited interactions can still occur between MtrC_DI,II_ and OmcA, but not between MtrAB and soluble OmcA.

**FIGURE 5:**
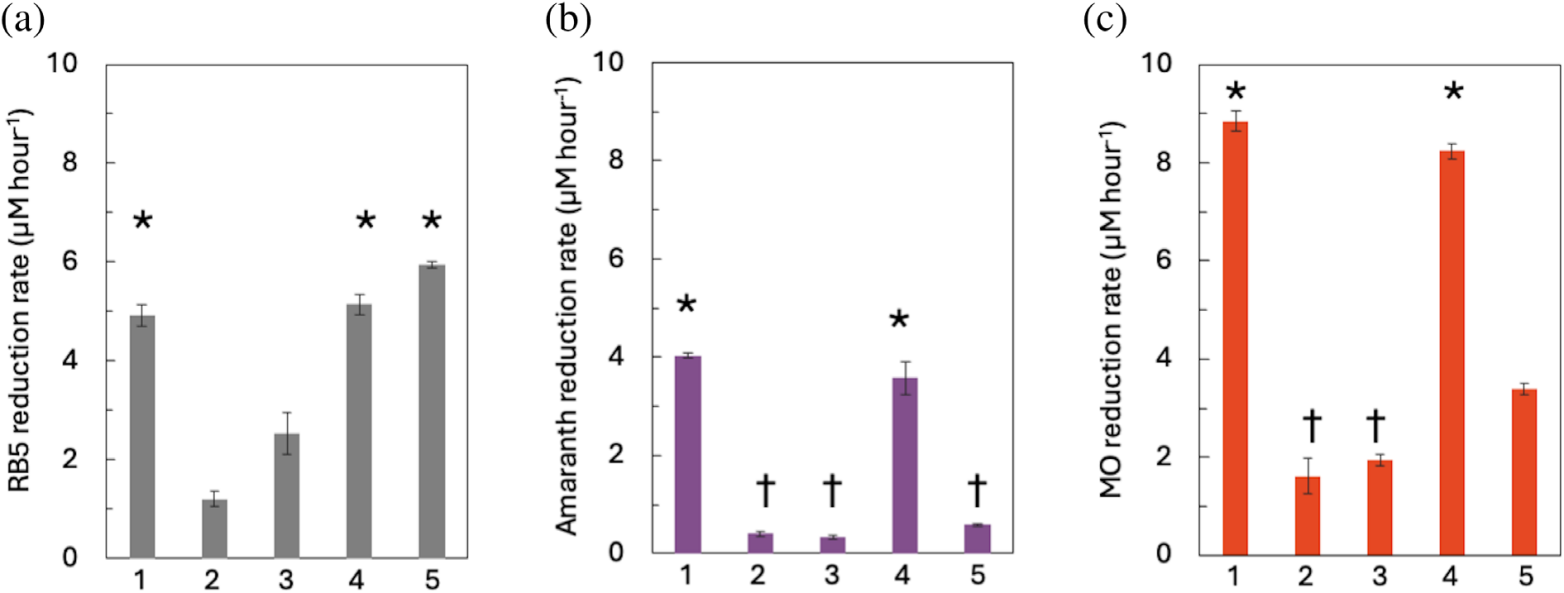
Cellular reduction of synthetic azo dyes. *Shewanella oneidensis* strains MR‐1 (1), ∆*mtr* (2), ∆*mtrC/omcA* (3), ∆*mtrC/omcA* MtrC_memb_ (4), and Δ*mtrC/omcA* MtrC_DI,II,memb_ (5) were incubated with Reactive Black 5 (a), Amaranth (b), and Methyl Orange (c). All assays performed with cells at a starting OD_600_ of 0.1. Rates calculated from change of absorbance over a period of 6 h. Experiments performed in triplicate and error bars show standard error of mean. Instances in which the rates did not differ significantly (*p* < 0.05) are indicated by either * or †, with each symbol representing a distinct data set.

While it was clear that *S. oneidensis* cells containing MtrC_DI,II_AB were unable to reduce FMN and Fe(III) citrate, it was not clear whether this was because the MtrC_DI,II_AB complex was incapable of electron transfer between MtrA and MtrC_DI,II_, or if the remaining MtrC domains I and II were limited in substrate reduction. To determine this, the synthetic azo dyes Amaranth, Methyl Orange, and RB5 were screened for their ability to be reduced by the different *S. oneidensis* strains (Figure 5).

The rate of RB5 reduction by *S. oneidensis* Δ*mtrC/omcA* MtrC_DI,II,memb_ was similar to both *S. oneidensis* MR‐1 and *S. oneidensis* Δ*mtrC/omcA* MtrC_memb_, showing that both the MtrC_DI,II_ and MtrC are capable of RB5 reduction using electrons delivered by MtrAB. Both *S. oneidensis* Δ*mtr* and *S. oneidensis* Δ*mtrC/omcA* showed substantially lower rates of RB5 catalytic reduction compared to strains containing an MtrC variant (Figure 5a).

The strain‐dependent reduction of Amaranth and Methyl Orange was similar to the rates of reduction of FMN or Fe(III) citrate, with strains unable to express full‐length MtrC showing a substantial decrease in catalytic reduction of Amaranth (Figure 5b). Methyl Orange was also reduced substantially faster in strains containing full‐length MtrC, although the *S. oneidensis* Δ*mtrC/omcA* MtrC_DI,II,memb_ strain showed a slight increase in catalytic activity when compared to strains lacking *omcA/mtrC* genes (Figure 5c).

## 4 DISCUSSION

The role and mechanism of OMCs in the reduction of extracellular electron acceptors has been the subject of intense discussion for many years. The *S. oneidensis* MR‐1 OMCs directly reduce soluble metals, lanthanides, and metal chelates, often resulting in the formation of metal precipitates at the cell surface (Rajput et al., 2021). Insoluble metal oxides can be both reduced directly or by secreted flavins that are reduced by the OMCs. The reduced flavins may mediate electron transfer between the cell and mineral, form electrotactic gradients that direct cells to mineral surfaces, or generate reactive flavocytochromes at the cell surface (Edwards et al., 2015; Norman et al., 2023; Shi et al., 2012).

**FIGURE 6:**
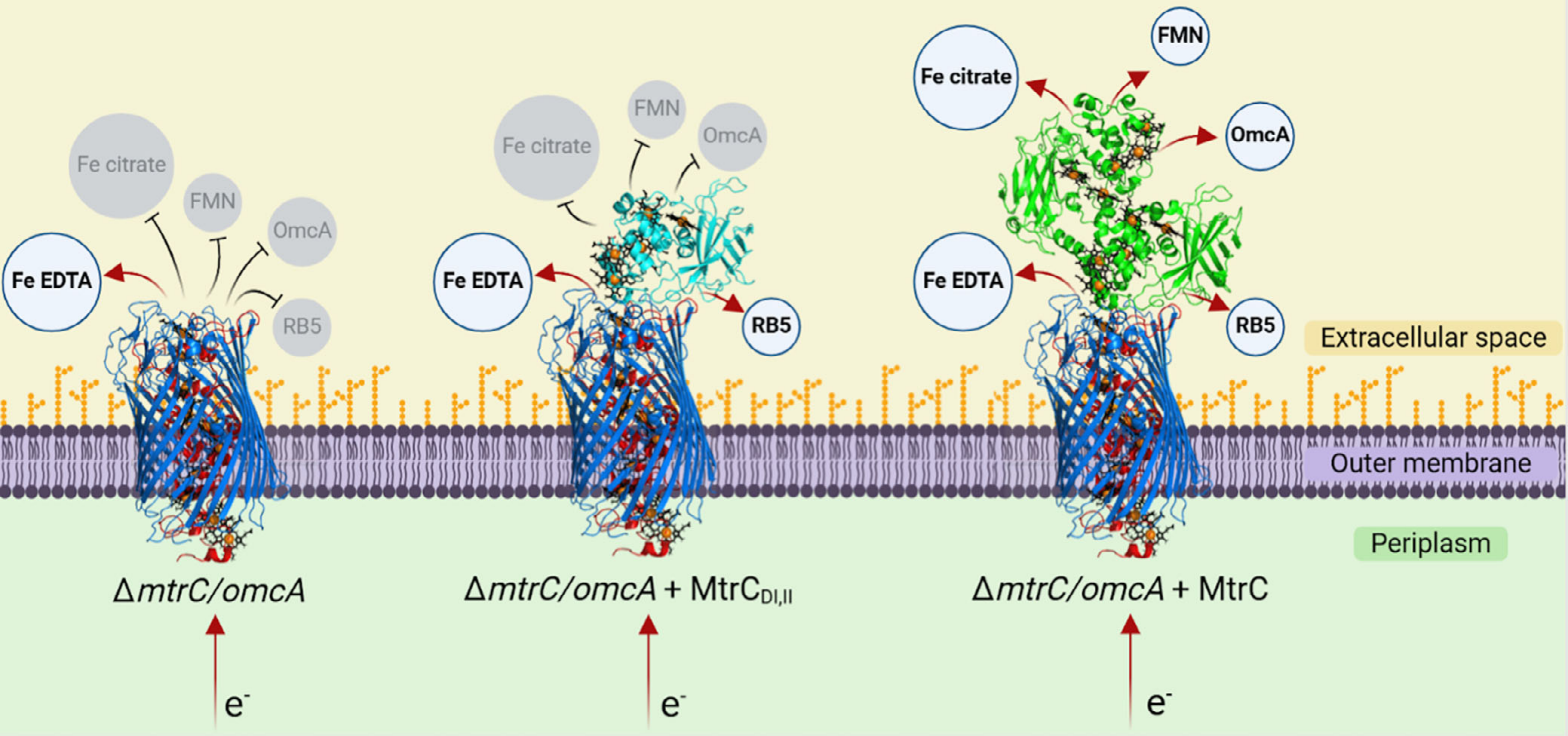
Summary of electron transfer through MtrAB, MtrC_DI,II_AB, and MtrCAB to external electron acceptors. Red arrows indicate direction of electron transfer. Black lines with T‐shaped arrowheads indicate no electron transfer. Blue circles with bold black text indicate acceptors where electron transfer occurred. Gray circles with gray text indicate acceptors where electron transfer did not occur. MtrA is shown in red, MtrB in blue, MtrC_DI,II,sol_ in cyan, and MtrC in green. MtrA, MtrB, and MtrC, are part of the *Shewanella baltica* MtrCAB crystal structure (PDB: 6R2Q). MtrC_DI,II,sol_ is from the *Shewanella oneidensis* crystal structure (PDB: 9EOV) Hemes are shown in black with the iron center as an orange sphere. Figure created using BioRender. FMN, flavin mononucleotide; RB5, Reactive Black 5.

In our experiments, the addition of a stop codon to *mtrC* resulted in the assembly of a truncated MtrC_DI,II_ variant, with domains I and II both structurally and spectroscopically identical to the corresponding domains of the full‐length protein. This MtrC_DI,II_ could interact with MtrAB, resulting in the formation of a 15‐heme MtrC_DI,II_AB nanowire capable of reducing a limited number of substrates, but unable to reduce the key physiological substrates FMN and Fe(III) citrate (Figure 6).

This data is in agreement with previous studies showing MtrC as the primary OMC responsible for both Fe(III) citrate and FMN reduction (Coursolle & Gralnick, 2010; Edwards et al., 2015; Wang et al., 2008). For all substrates tested here, the recombinant expression of *mtrC* resulted in reduction rates equal to or greater than those observed for the wild type, while the *S. oneidensis* Δ*mtrC/omcA* strain could only reduce Fe(III) EDTA at rates similar to *S. oneidensis* MR‐1. The surface of *S. oneidensis* is covered by a heterogeneous lipopolysaccharide (LPS) layer that varies in size and coverage (Korenevsky et al., 2002). It is possible that this LPS may have prevented the access of FMN and Fe(III) citrate to either MtrAB or MtrC_DI,II_AB. However, RB5 (991.8 Da) is significantly larger than FMN (456.3 Da) and can still be reduced by MtrC_DI,II_AB, suggesting that both Fe(III) citrate and FMN are able to access the MtrC_DI,II_AB complex but cannot be reduced by this form.

Previous studies showed OmcA was able to reduce both FMN and Fe(III) citrate, although at rates much lower than when MtrC was produced, indicating that it was possible for electrons to pass to OmcA from MtrAB (Coursolle & Gralnick, 2010). In the experiments described here, cells producing MtrAB were unable to reduce soluble OmcA. Previously we showed that MtrC_sol_ added to *S. oneidensis* Δ*mtrC* cells formed a stable MtrCAB complex on the outer membrane, indicating that even large OMCs are not restricted from the cell surface by the LPS (Lockwood et al., 2018). This suggested that the membrane anchor was important for MtrAB‐OmcA interactions, likely by keeping OmcA localized to the outer leaflet of the lipid bilayer. While a stable MtrCAB‐OmcA complex has never been observed, MtrC was able to efficiently reduce OmcA, suggesting that there were specific interaction sites between OmcA and MtrC that facilitated electron exchange. The loss of OmcA reduction activity observed in cells containing MtrC_DI,II_AB supports this and indicates that domains III and IV contain the site of OmcA reduction.

The “staggered cross” arrangement of the hemes within MtrC is highly conserved in all *Shewanella* OMCs. Electrons are passed to MtrC heme 5 from MtrA and can rapidly travel through all 10 closely packed hemes (Figure 1b). Heme 10 in domain IV is the furthest heme from the electron ingress site and appears well‐positioned to transfer electrons to substrates adjacent to the cell. In addition, previous *in silico* modeling predicted that a FMN binding site might exist near heme 7 of MtrC domain IV (Breuer et al., 2015). This site is close enough for electron transfer, with the isoalloxazine ring positioned between domains III and IV. Our results support these findings by revealing MtrC contains specific catalytic sites for OmcA, FMN, and Fe(III) citrate that require the presence of domains III and IV.

While it is not usual for multiheme cytochromes to contain chains of hemes leading to a terminal active site, the arrangement of hemes across domains II and IV is unusual. It is possible that this interdomain electron transfer pathway provides an important kinetic purpose: rearrangement of domains could form a new MtrC conformation where the hemes at the domain II/IV interface are no longer within viable electron transfer distance. In agreement with this, the helix that connects domains II and III contains a kink that could support movement of the two MtrC halves and provides a possible mechanism to facilitate the dislocation of the domain II/IV electron transport pathway (Edwards et al., 2020). The efficient reduction of FMN by *S. oneidensis* can be dangerous on exposure to oxygen, as the reduced flavins can generate cytotoxic hydrogen peroxide. To limit this effect, a cysteine pair on the MtrC surface forms a disulfide bond in the presence of oxygen which arrests flavin reduction (Edwards et al., 2015). It has been suggested that the formation of the MtrC disulfide causes a repositioning of the heme domains on the cell surface that disrupts the electron transfer pathway between the two pentaheme MtrC domains (Norman et al., 2023). The *in vivo* catalytic experiments presented here demonstrate that the reduction of physiological substrates requires the full 4‐domain structure of MtrC, yet reduction of certain other substrates is still achievable via the 2‐domain MtrC.

## AUTHOR CONTRIBUTIONS

**Alejandro Morales‐Florez:** Conceptualization; investigation; methodology; writing – review and editing; validation; writing – original draft. **Colin W. J. Lockwood:** Conceptualization; investigation; methodology. **Benjamin W. Nash:** Investigation; methodology; data curation. **Marcus J. Edwards:** Supervision; conceptualization; investigation; validation; writing – review and editing. **Jessica H. van Wonderen:** Investigation; validation; methodology. **Amit Sachdeva:** Investigation; conceptualization; validation; supervision. **Julea N. Butt:** Conceptualization; investigation; validation; writing – review and editing; funding acquisition; formal analysis; supervision. **Thomas A. Clarke:** Conceptualization; investigation; visualization; validation; writing – review and editing; funding acquisition; writing – original draft; formal analysis; project administration; supervision.

## CONFLICT OF INTEREST STATEMENT

The authors declare no competing interests.

## Supporting information


**Data S1.** Supporting Information.

## Data Availability

The data that support the findings of this study are openly available in Figshare at https://figshare.com/, reference number 10.6084/m9.figshare.29516924.
