# Supplementary material for: Extracellular catalysis of environmental substrates by Shewanella oneidensis MR‐1 occurs via active sites on the C‐terminal domains of MtrC
Source: Protein Sci. 2025 Jul 28;34(8):e70243. doi: 10.1002/pro.70243 (PMC12304082; doi:10.1002/pro.70243)
Supplement: Supplementary file 1 — Data S1. Supporting Information. [file PRO-34-e70243-s001.docx]

Extracellular Microbial Catalysis of Environmental Substrates by *Shewanella oneidensis* MR-1 occurs via active sites located on domains III and IV of MtrC

**Alejandro Morales-Florez^1^, Colin W. J. Lockwood^2^, Benjamin W. Nash^1^, Marcus J. Edwards^3^, Jessica H. van Wonderen^1^, Amit Sachdeva^2^, Julea N. Butt^1,2^, Thomas A. Clarke^1,4^**

**SUPPLEMENTARY INFORMATION**

**Table S1. Strains and Plasmids used in this manuscript.**

| **Strains** | | | **Relevant Feature** | **Source** | |  |
| --- | --- | --- | --- | --- | --- | --- |
| *Shewanella oneidensis* | MR-1 | | Wild type | Lab stock | |  |
|  | Δ*mtrC/omcA* | | MR-1 with genomic *mtrC* and *omcA* knocked out | Meitl *et al.* [1] | |  |
|  | Δ*mtr* | | MR-1 with *mtr* cluster knocked out | Edwards *et al*. [2] | |  |
|  | Δ*mtrC/omcA* MtrC_memb_ | | MR-1 with genomic *mtrC* and *omcA* knocked out, containing **pLS138** and producing **MtrC_memb_** | This manuscript | |  |
|  | Δ*mtrC/omcA* MtrC_DI,II, sol_ | | MR-1 with genomic *mtrC* and *omcA* knocked out, containing **pAMF1** and producing **MtrC_DI,II, sol_** | This manuscript | |  |
|  | Δ*mtrC/omcA* MtrC_DI,II, memb_ | | MR-1 with genomic *mtrC* and *omcA* knocked out, containing **pAMF2** and producing **MtrC_DI,II,memb_** | This manuscript | |  |
|  | Δ*mtr* MtrC_memb_ | | MR-1 with *mtr* cluster knocked out, containing **pLS138** and producing **MtrC_memb_** | This manuscript | |  |
|  | Δ*mtr* MtrC_DI,II, memb_ | | MR-1 with *mtr* cluster knocked out, containing **pAMF2** and producing **MtrC_DI,II, memb_** | This manuscript | |  |
|  |  | |  |  | |  |
|  | | |  |  | |  |
| **Plasmids** | | **Relevant** **Feature** | | | **Source/Reference** | |
| pJvW001 | | pBAD/TOPO plasmid encoding for a soluble full-length MtrC with *mtrB* signal peptide and a C-terminus Strep(II) tag, **MtrC_sol_** | | | Lockwood *et al.* [3] | |
| pLS138 | | pBAD/TOPO plasmid encoding for a lipid-anchored full-length MtrC with the native *mtrC* signal peptide, **MtrC_memb_** | | | Shi *et al*. [4] | |
| pAMF1 | | pBAD/TOPO plasmid encoding for a soluble domain I and II MtrC with *mtrB* signal peptide and glutamic acid codon at amino acid position 344 changed to an amber stop codon, **MtrC_DI,II, sol_** | | | This manuscript, and Lockwood *et al.*[5] | |
| pAMF2 | | pBAD/TOPO plasmid encoding for a lipid-anchored domain I and II MtrC with the native *mtrC* signal peptide with glutamic acid codon at amino acid position 344 changed to an amber stop codon, **MtrC_DI,II, memb_** | | | This manuscript | |

**Table S2. Primers used in this manuscript.** Stop codon is indicated in **bold**.

| **Primers** | **Sequence (5’ → 3’)** | **Description** |
| --- | --- | --- |
| *mtrC*_E344TAG_ forward | CAATTAATACC**TAG**ACTAAAGCAG | Primers for introduction of Amber stop codon at position E344 in pJvW001 and pLS138 |
| *mtrC*_E344TAG_ reverse | CTGCTTTAGT**CTA**GGTATTAATTG |  |

**Table S3. Data collection and refinement statistics for MtrC domains I and II crystal structure.**

| MtrC domains I, II | |
| --- | --- |
| Data collection | |
| Space group | P 2_1_ 2_1_ 2­_1_ |
| Cell dimensions |  |
| *a*, *b*, *c* (Å) | 74.28 77.35 96.53 |
| α, β, γ (°) | 90.00, 90.00, 90.00 |
| Resolution (Å) | 58.87 - 1.80 (1.86 - 1.80) |
| *CC_1/2_* (%) | 99.4 (80.2) |
| *I / σI* | 6.92 (0.63) |
| Completeness (%) | 99.8 (99.9) |
| Multiplicity | 12.5 (12.7) |
| Refinement | |
| Resolution (Å) | 1.80 |
| No. reflections | 52134 |
| *R*_work_ / *R*_free_ | 0.190/0.221 |
| No. atoms |  |
| Protein | 2128 |
| Ligand/ion | 228 |
| Water | 367 |
| *B*-factors |  |
| Protein | 29.03 |
| Ligand/ion | 23.04 |
| Water | 39.30 |
| R.m.s. deviations |  |
| Bond lengths (Å) | 0.020 |
| Bond angles (°) | 2.06 |

**Table S4. Processed results from sedimentation velocity experiments.** The deconvoluted diffusion sedimentation coefficient distribution and frictional coefficients show differences between MtrAB, MtrC_DI,II_AB, and MtrCAB. In the MtrAB sample, MtrAB accounted for 99% of the experimental mass, sedimentation coefficient and frictional coefficient. For the MtrC_DI,II_AB sample, MtrC_DI,II_AB accounted for 92% of the experimental mass, sedimentation coefficient, and frictional coefficient. For the MtrCAB sample, MtrCAB accounted for 86% of the experimental mass, sedimentation coefficient, and frictional coefficient. The partial specific volume for all samples was 0.72 mg mL^-1^. All samples were in 20 mM HEPES, 150 mM NaCl, 5 mM LDAO, pH 7.8.

| **Sample** | **Predicted**  **mass (kDa)** | **Experimental mass (kDa)** | **Sedimentation**  **coefficient (S)** | **Frictional**  **ratio** $\left( \frac{\boldsymbol{f}}{\boldsymbol{f}_{\boldsymbol{0}}} \right)$ |
| --- | --- | --- | --- | --- |
| MtrAB | 114 | 96 ± 4 | 4.8 | 1.55 |
| MtrC_DI,II_AB | 149 | 144 ± 14 | 5.2 | 1.86 |
| MtrCAB | 185 | 199 ±15 | 7.4 | 1.56 |

**Figure S1. Identification of purified MtrC_DI,II, sol_.** Heme-stained SDS-PAGE gels showing fractions after elution from a DEAE anionic exchange chromatography column **(A)**, and a size-exclusion chromatography (SEC) column **(B)** from the MtrC_DI,II, sol_ protein purification. Molecular markers (kDa) are labelled on the leftmost lane. FccA is almost entirely removed after the SEC, and the sample is largely composed of soluble MtrC_DI,II_.

A

B


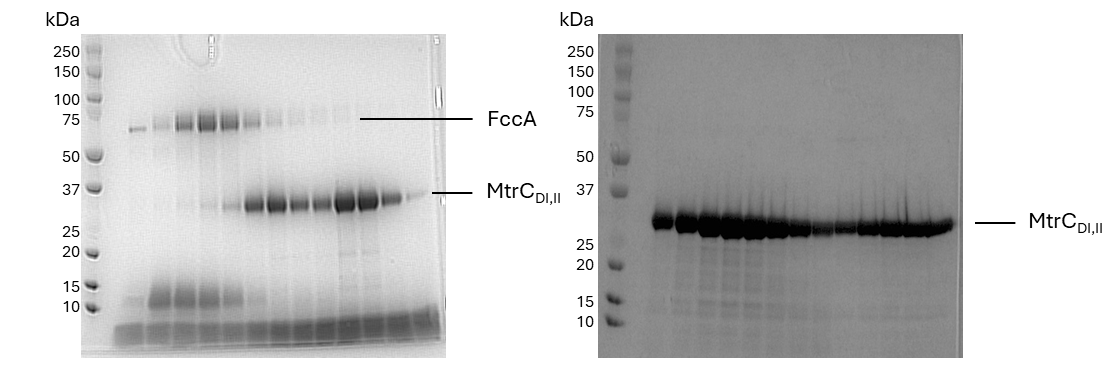


**Figure S2. MtrC_DI,II, sol_ LC-MS data analysis showing the detected intensity units as a function of molecular weight.** The maximum value for the intensity is shown as a data label on the graph, and corresponds to the experimental molecular weight of MtrC_DI,II, sol_.
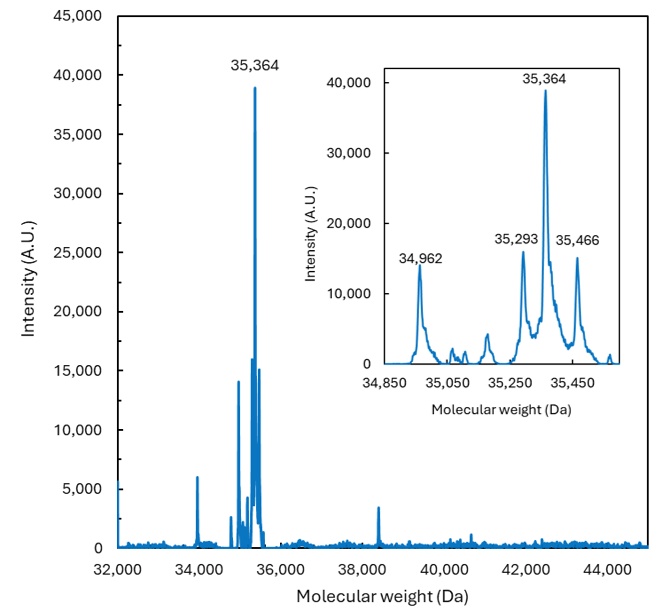
Inset: adjacent intensity peaks are also labelled.

**Figure S3. MtrC_DI,II_ crystal structure and models. (A)**. Crystal structure of MtrC_DI,II, sol_ resolved to residue A321 at 1.8 Å (PDB: 9EOV). **(B)**. MtrC_DI,II, sol_ crystal structure (cyan) superposed with the crystal structure of MtrC from *S. oneidensis* MR-1 (green; PDB: 4LM8) with an RMSD of 0.380 Å. Relevant amino acid residues are shown in colour: the C-terminal residue is tyrosine 343 (red); the C-terminal residue observed by LC-MS is alanine 325 (magenta); and the C-terminal residue resolved by X-ray crystallography is alanine 321, shown in orange in shown in MtrC_DI,II, sol_ and dark green in MtrC.**(C)**. Magnified view of the C-terminal residues observed by LC-MS and X-ray crystallography. **(D)**. MtrC_DI,II, sol_ crystal structure (cyan) superposed with the crystal structure for MtrCAB from *S. baltica* OS185 (PDB:6R2Q) with an RMSD of 1.029 Å. *S. baltica* MtrC is shown in green, MtrA in red, and MtrB in blue. Crystal structures and models visualised using PyMOL Molecular Graphics System, version 3.0 (Schrödinger, LLC). Hemes are numbered in blue, according to the position of the heme binding motif within the amino acid chain.

C


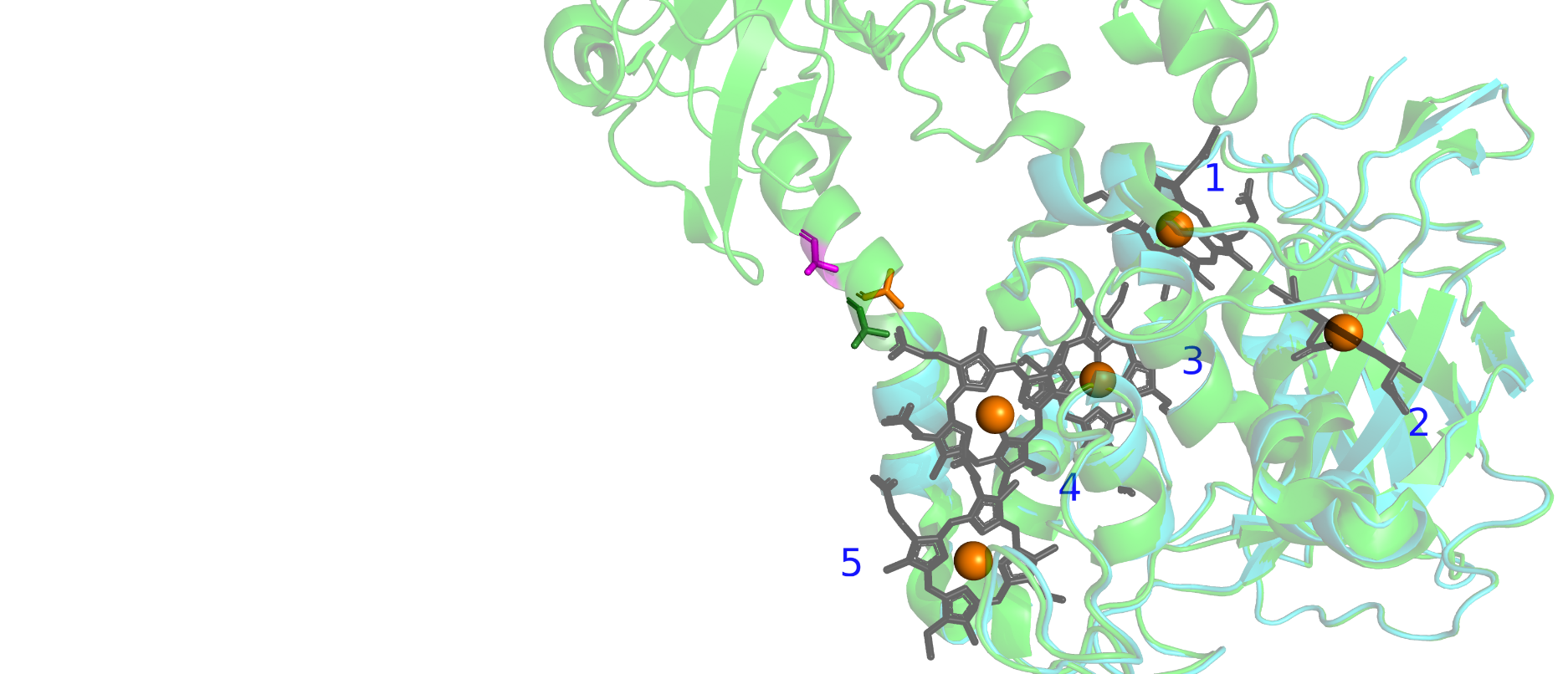

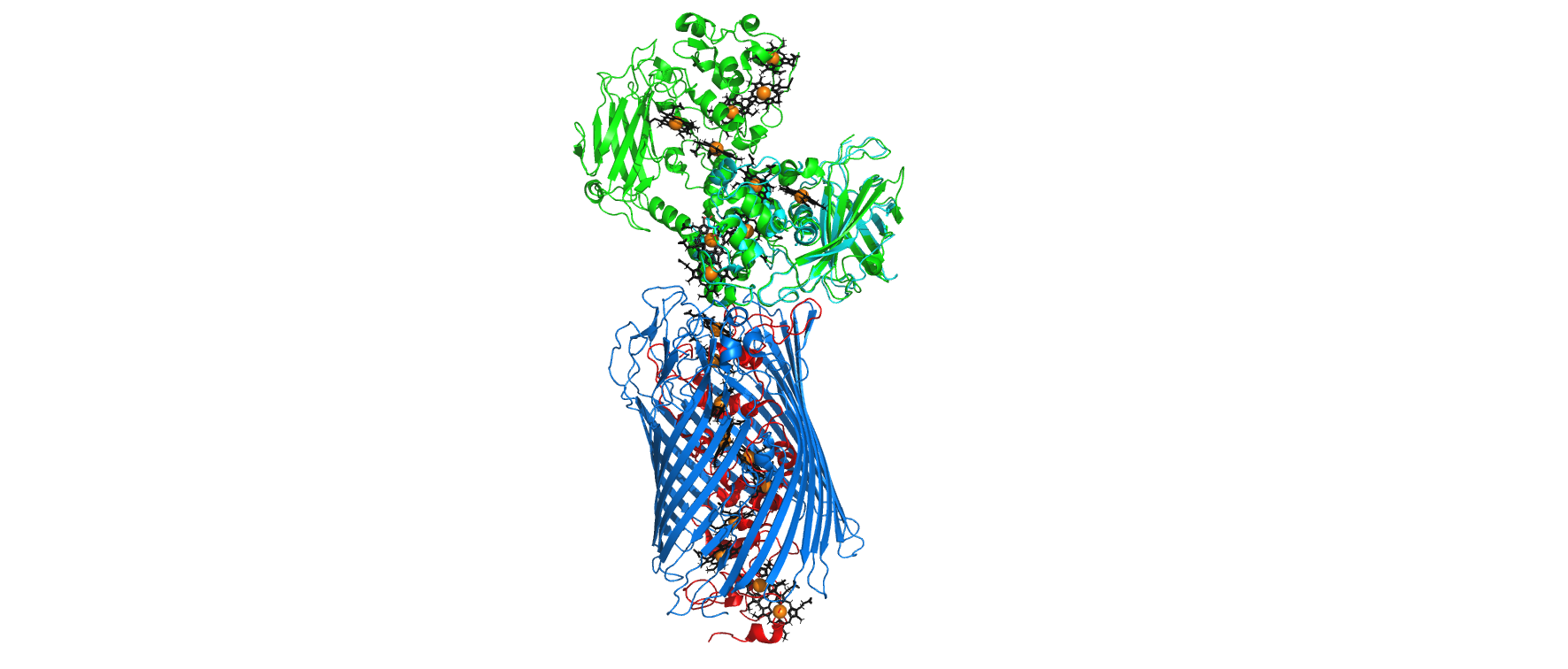


D


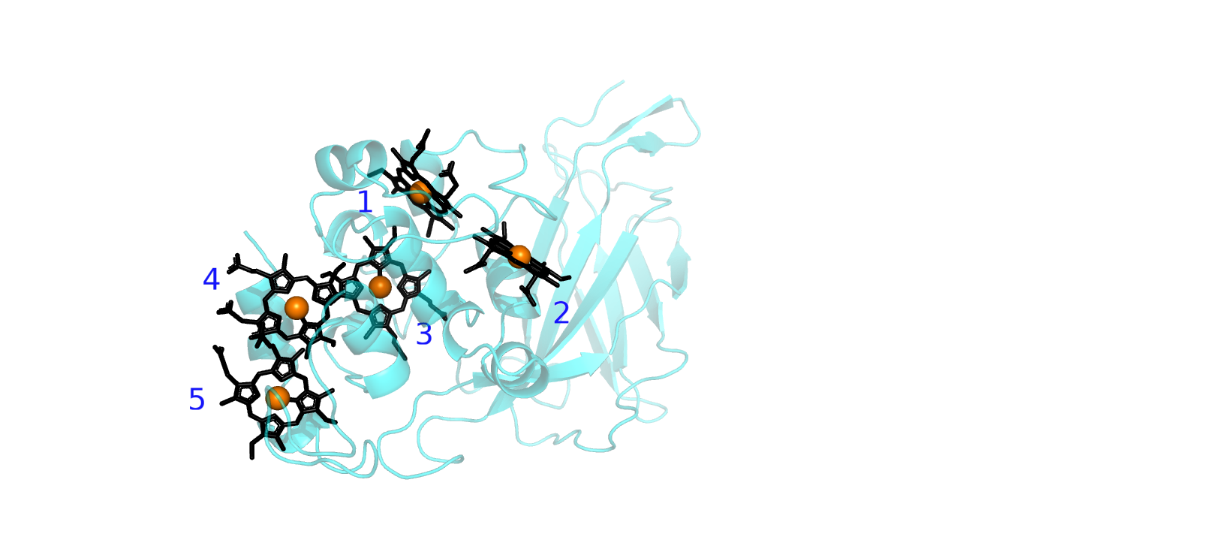


A

B


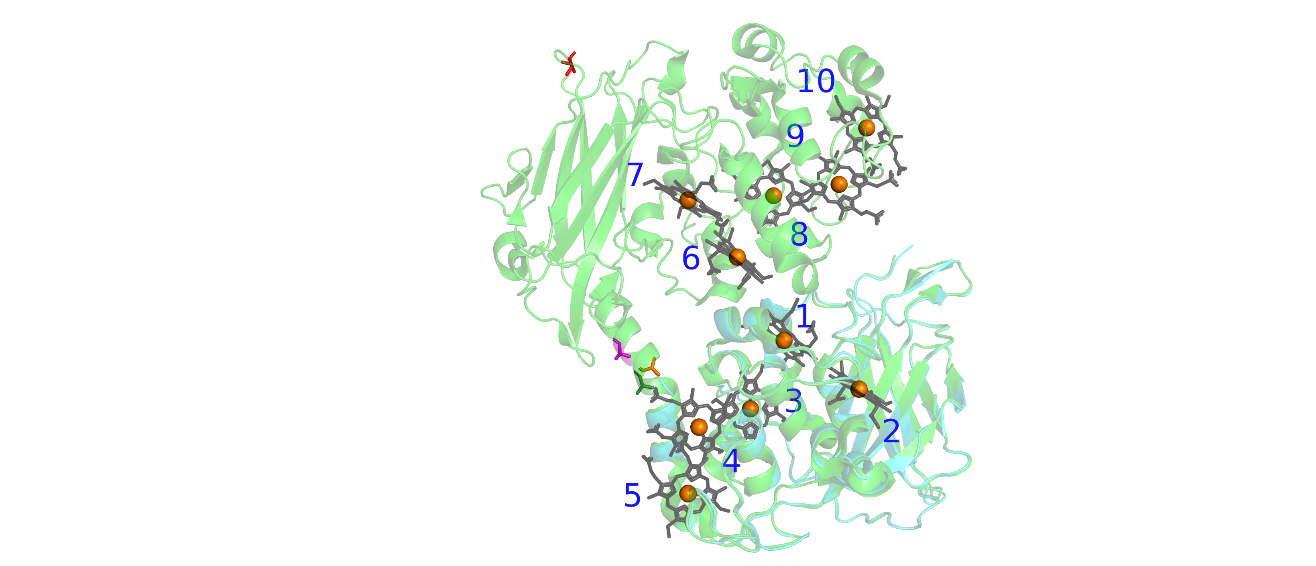


References

1. Meitl LA, Eggleston CM, Colberg PJS, Khare N, Reardon CL, Shi L. Electrochemical interaction of *Shewanella oneidensis* MR-1 and its outer membrane cytochromes OmcA and MtrC with hematite electrodes. *Geochim Cosmochim Acta* 2009; **73**: 5292–5307.

2. Edwards MJ, White GF, Butt JN, Richardson DJ, Clarke TA. The Crystal Structure of a Biological Insulated Transmembrane Molecular Wire. *Cell* 2020; **181**: 665-673.e10.

3. Lockwood CWJ, van Wonderen JH, Edwards MJ, Piper SEH, White GF, Newton-Payne S, et al. Membrane-spanning electron transfer proteins from electrogenic bacteria: Production and investigation. *Methods Enzymol* 2018; **613**: 257–275.

4. Shi L, Deng S, Marshall MJ, Wang Z, Kennedy DW, Dohnalkova AC, et al. Direct involvement of type II secretion system in extracellular translocation of *Shewanella oneidensis* outer membrane cytochromes MtrC and OmcA. *J Bacteriol* 2008; **190**: 5512–5516.

5. Lockwood CWJ, Nash BW, Newton-Payne SE, van Wonderen JH, Whiting KPS, Connolly A, et al. Genetic Code Expansion in *Shewanella oneidensis* MR-1 Allows Site-Specific Incorporation of Bioorthogonal Functional Groups into a *c* -Type Cytochrome. *ACS Synth Biol* 2024.
